# Percutaneous stent placement for the treatment of malignant biliary obstruction: nitinol versus elgiloy stents

**DOI:** 10.1590/0100-3984.2015.0183

**Published:** 2017

**Authors:** Charles Edouard Zurstrassen, Almir Galvão Vieira Bitencourt, Marcos Duarte Guimaraes, Aline Cristine Barbosa Santos Cavalcante, Chiang Jeng Tyng, Mauricio Kauark Amoedo, João Paulo Kawaoka Matsushita Junior, Janio Szklaruk, Edson Marchiori, Rubens Chojniak

**Affiliations:** 1 Head of the Department of Interventional Radiology, A.C.Camargo Cancer Center, São Paulo, SP, Brazil; 2 Staff Physician in the Department of Imaging, A.C.Camargo Cancer Center, São Paulo, SP, Brazil; 3 Staff Physician in the Department of Imaging, A.C.Camargo Cancer Center, São Paulo, SP, Assistant Professor of Radiology, Universidade Federal do Vale do São Francisco (UNIVASF), Petrolina, PE, Brazil; 4 Staff Physician in the Department of Interventional Radiology, A.C.Camargo Cancer Center, São Paulo, SP, Brazil; 5 Professor of Radiology, Department of Diagnostic Radiology, Division of Diagnostic Imaging, The University of Texas MD Anderson Cancer Center, Houston, TX, USA; 6 Full Professor of Radiology, Universidade Federal do Rio de Janeiro (UFRJ), Rio de Janeiro, RJ, Brazil; 7 Head of the Department of Imaging, A.C.Camargo Cancer Center, São Paulo, SP, Brazil

**Keywords:** Radiology, interventional, Drainage, Stents, Biliary tract/pathology, Oncology

## Abstract

**Objective:**

This study aimed to compare two self-expanding stents, a nitinol stent and an elgiloy stent, both placed percutaneously, in terms of their efficacy in palliating inoperable malignant biliary obstruction.

**Materials and Methods:**

We retrospectively investigated 99 patients with unresectable malignant biliary obstruction treated with percutaneous placement of a self-expanding metallic stent at our institution between May 2007 and January 2010. Serum bilirubin and liver enzyme levels were measured before and 30 days after stenting. For all procedures using elgiloy or nitinol stents, stent occlusion and patient survival rates were calculated using Kaplan-Meyer analysis.

**Results:**

All of the patients showed clinical improvement after stent placement, with no difference between the two groups. In both groups, the occlusion-free survival rate was 67% at 30 days, 37% at 90 days, 25% at 180 days, and 10% at 360 days, with no significant difference in relation to the type of stent.

**Conclusion:**

The two stents evaluated showed comparable efficacy for the percutaneous treatment of unresectable biliary malignancy, with good clinical results.

## Resumo

**Objetivo:**

Este estudo procurou comparar a eficácia do implante percutâneo do *stent* autoexpansível de nitinol *versus* o *stent* de elgiloy para paliação da obstrução biliar maligna irressecável.

**Materiais e Métodos:**

Nós investigamos, retrospectivamente, 99 pacientes com obstrução maligna irressecável tratada com implante percutâneo de *stent* metálico autoexpansível em nossa instituição, de março de 2007 até janeiro de 2010. Os níveis séricos de bilirrubina e enzimas hepáticas foram medidos antes e 30 dias após o implante do *stent*. Para os procedimentos em que foi utilizado o *stent* de elgiloy ou o *stent* de nitinol, as taxas de oclusão dos *stents* e as taxas de sobrevida dos pacientes foram calculadas pela análise de Kaplan-Meyer.

**Resultados:**

Os pacientes mostraram melhora clínica após o implante dos *stents*, sem diferença entre os dois grupos. As taxas de sobrevida livre de oclusão foram 67% aos 30 dias, 37% aos 90 dias, 25% aos 180 dias e 10% aos 360 dias para ambos os grupos, sem diferença significativa em relação ao tipo de *stent*.

**Conclusão:**

Os dois stents mostraram eficácias comparáveis no tratamento da doença biliar maligna irressecável, com bons resultados clínicos.

## INTRODUCTION

Biliary tract obstruction is an important adverse factor for the treatment of patients with unresectable tumors involving the intrahepatic or extrahepatic bile ducts^(1)^. Percutaneous or endoscopic biliary drainage and stenting has been the standard palliative treatment in these cases^(2)^, and improved therapeutic regimens have extended the life expectancy of patients. Therefore, considerable efforts have been made in order to reduce the number of complications associated with stent implantation, especially stent occlusion, a major complication that can be difficult to manage^(1-3)^.

Self-expanding metallic stents have provided better results than have plastic stents in relation to the occlusion rate and medium- to long-term cost-effectiveness^(4,5)^. However, even metallic stents may become occluded, mainly because of tumor ingrowth through the stent mesh^(6)^. Therefore, various types of metallic stents, made of a wide variety of materials and with different mesh characteristics, have been developed in attempt to prevent stent occlusion^(7-11)^.

This study aimed to compare the efficacy of two self-expanding stents-a bare nitinol stent (S.M.A.R.T. CONTROL; Cordis Corp. Miami, FL, USA) and an elgiloy stent (Wallstent; Boston Scientific, Watertown, MA, USA), both placed percutaneously, in the palliative treatment of inoperable malignant biliary obstruction.

## MATERIALS AND METHODS

### Study design and patients

The study was a retrospective clinical investigation. All patients undergoing percutaneous treatment of malignant biliary obstruction at our institution between May 2007 and January 2010 were reviewed for eligibility. The study was approved by the research ethics committee of the institution and conducted in accordance with the provisions of the Declaration of Helsinki. At the time of the procedure, all patients gave written informed consent for future use of their data.

The inclusion criteria for this study were as follows: diagnosis of malignant biliary obstruction confirmed by percutaneous or surgical biopsy; unresectable disease; and percutaneous placement of a dedicated self-expanding metallic stent for the treatment of biliary disease. Unresectability was defined as the presence of vascular invasion, extrahepatic disease, or medical comorbidities that prevented surgical liver resection. The site of biliary obstruction was divided into periampullary and hilar regions, and hilar strictures were subdivided using the Bismuth classification^(12)^.

The following exclusion criteria were applied: resectable disease; ascites; uncontrollable coagulopathy (international normalized ratio > 3.0); history of a serious allergic reaction to iodinated contrast media; and previous biliodigestive anastomosis.

### Stent placement

The procedures, all of which were performed when the patients were under local anesthesia and sedation, were guided by percutaneous transhepatic cholangiography. Antibiotic prophylaxis was given in all cases. Patients with a previous diagnosis of cholangitis were being treated with antibiotics based on bile culture and sensitivity.

Cholangiography was performed using fluoroscopy (Axiom Artis; Siemens, Erlangen, Germany), and a percutaneous transhepatic puncture was performed with a 22-gauge Chiba needle. When a puncture to the left hepatic lobe was necessary, ultrasound guidance was used.

The bile ducts were punctured on either the right or left side when the stricture was located in the periampullary region or in patients with Bismuth type I hilar strictures. The bile ducts were punctured on both the right and left sides in patients with Bismuth type II hilar strictures. When biliary obstruction involved right or left segments of secondary bile ducts (Bismuth III and IV strictures), more than one puncture of the liver lobes involved was performed.

In the presence of cholangitis or when it was not possible to advance the hydrophilic guide wire across the stricture, a temporary external drainage catheter was placed (Ultrathane^®^; Cook Medical, Inc., Bloomington, IN, USA). After an observation period of 3–5 days, patients with drainage catheters returned to the operating room for treatment completion (secondary stenting technique). In patients without evidence of cholangitis and in whom the guide wire could be advanced through the stricture to the duodenal loop, a primary stenting technique was performed with direct placement of the stent.

Self-expanding metallic stents were chosen according to their availability in the operating room. When a residual stenosis that prevented the contrast medium from flowing into the duodenal loop was observed, the stricture was dilated with a semi-compliant balloon catheter (Powerflex; Cordis Corp., Miami, FL, USA).

Only one stent was used in patients with periampullary lesions or Bismuth type I hilar strictures. In patients with Bismuth type II hilar strictures, two stents were often employed. In patients with Bismuth type III or IV hilar strictures, up to three stents were used in an attempt to drain the largest possible number of obstructed liver segments.

After the success of the procedure was confirmed by proper clearance of the contrast medium injected into the intrahepatic bile ducts, the introducer sheath was removed and the patient was transferred to the recovery room.

### Follow-up

Clinical evaluation and laboratory assessment of liver enzymes (aspartate aminotransferase and alanine aminotransferase), canalicular enzymes (alkaline phosphatase and gamma-glutamyl transpeptidase), and serum (total and direct) bilirubin levels were performed before and 30 days after the procedure. Patients were followed up for assessment of survival and presence of stent occlusion at 30, 90, 180, and 360 days. Stent occlusion was defined as recurrence of biliary obstruction confirmed by imaging studies and laboratory tests (serum bilirubin levels > 3 mg/dL).

Complications such as bleeding, cholangitis, cholecystitis, and pancreatitis were diagnosed on the basis of the clinical signs and symptoms, together with the results of laboratory tests, as well as those of imaging studies, when indicated. In case of the recurrence of jaundice, magnetic resonance imaging or computed tomography of the liver was performed to determine whether jaundice was caused by advanced metastatic disease or the intrahepatic ducts were dilated and reintervention was required. We defined minor complications as events requiring nominal therapy or observation without sequelae and major complications as events requiring patient hospitalization. Any complication or death occurring within 30 days of stent insertion was considered procedure-related.

### Statistical analysis

Patients and procedures were characterized using descriptive statistics. Continuous variables were expressed as mean ± standard deviation, and categorical variables were expressed as absolute and relative frequencies. In the comparative analysis, we included only those procedures in which selfexpanding nitinol S.M.A.R.T. CONTROL stents or elgiloy Wallstents were used. In comparisons between the S.M.A.R.T. CONTROL and Wallstent groups, the Student's *t*-test was used in order to compare mean patient ages, and the chi-square test was used in order to compare the levels of biliary obstruction. Biochemical values obtained before and after stent placement were compared, and the differences were analyzed by nonparametric repeated-measures analysis of variance.

For the S.M.A.R.T. CONTROL and Wallstent groups, stent occlusion and patient survival rates were calculated using Kaplan-Meyer survival (life-table) analysis. The logrank test was used to assess the differences in occlusion-free progression between the two groups. Survival curves were compared between the two groups using a Cox proportional hazards model adjusted for age and level of biliary obstruction. Data were analyzed using the IBM SPSS Statistics, version 20.0 (IBM Corporation, Armonk, NY, USA). Values of *p* < 0.05 were considered statistically significant.

## RESULTS

Between May 2007 and January 2010, 99 patients with unresectable malignant biliary obstruction underwent percutaneous placement of a self-expanding metallic stent at our institution. Of those 99 patients, 1 (0.9%) underwent three procedures, 8 (7.0%) underwent two procedures, and 90 (92.1%) underwent only one procedure. Therefore, a total of 109 procedures were performed. The mean age of patients was 60.4 ± 12.1 years (range, 29–84 years), and 51% were men. Table 1 shows the causes of biliary tract obstruction among the patients in the sample.

**Table 1:** Distribution of the etiology of biliary tract obstruction.

Underlying diagnosis	N	%
Colorectal cancer (with lymph node metastases)	22	22.2
Gastric cancer (with lymph node metastases)	15	15.1
Cholangiocarcinoma	14	14.1
Cancer of the head of the pancreas	14	14.1
Gallbladder cancer	8	8.0
Pancreatic cancer (with lymph node metastases)	6	6.0
Breast cancer (with lymph node metastases)	5	5.2
Hepatocellular carcinoma	2	2.1
Ovarian cancer (with lymph node metastases)	2	2.1
Other	11	11.1
Total	99	100

Of the 109 procedures evaluated, 92 (84.4%) were performed in order to treat hilar strictures-classified as Bismuth type I in 33 cases (30.3%), as Bismuth type II in 19 (17.4%), as Bismuth type IIIA in 24 (22.0%), and as Bismuth type IIIB in 16 (14.7%)-the remaining 17 (15.6%) being performed in order to treat periampullary lesions. Percutaneous biliary drainage was performed before stent placement in 37 (33.9%) of the procedures. In 35 procedures (32.1%), bilateral drainage was performed. In 80 (73.4%) of the procedures, the biliary stricture was dilated with a balloon catheter after stent insertion.

A single stent was used in 66 (60.4%) of the procedures (all to treat periampullary or Bismuth I strictures), two stents were used in 36 (33.0%) of the procedures (all to treat Bismuth II strictures), and three stents were used in 7 (5.6%) of the procedures (all to treat Bismuth III and IV strictures). Wallstents were used in 61 procedures (56%), and S.M.A.R.T. CONTROL stents were used in 48 (44%).

The most common complication was acute pancreatitis, occurring in 31 cases (28.4%), followed by cholangitis, in 12 (11.0%), bronchopneumonia, in 5 (4.6%) thromboembolism, in 4 (3.7%), and transient hemobilia, in 3 (2.7%). Twenty-eight patients died within the first 30 days after the procedure, the overall 30-day mortality rate being 28.3%.

### Comparative analysis: Wallstents vs. S.M.A.R.T. CONTROL stents

The Wallstent group consisted of 58 patients, with a mean age of 59.4 ± 12.9 years (range, 29–84 years), who collectively underwent 61 procedures, and the S.M.A.R.T. CONTROL group consisted of 46 patients, with a mean age of 62.8 ± 10.7 years (range, 36–85 years), who collectively underwent 48 procedures. There was no statistically significant difference between the two groups regarding age (*p* = 0.136) or level of biliary obstruction (*p* = 0.685).

Table 2 shows the laboratory test results before and 30 days after stent placement in the S.M.A.R.T. CONTROL and Wallstent groups. Overall, patients showed post-treatment reductions in the mean values of biochemical variables. There was no statistically significant difference between the two groups in terms of the post-treatment reductions in mean (total and direct) bilirubin levels and mean levels of canalicular enzymes (alkaline phosphatase and gamma-glutamyl transpeptidase). However, the reductions in aspartate aminotransferase and alanine aminotransferase levels after stent placement were greater in the S.M.A.R.T. CONTROL group than in the Wallstent group (Table 2).

**Table 2:** Biochemical variables before and 30 days after stent placement in patients treated with percutaneous placement of self-expanding elgiloy (Wallstent) or nitinol (S.M.A.R.T. CONTROL) stents.

	Wallstent (*n* = 61)			S.M.A.R.T. CONTROL (*n* = 48)		
Laboratory values	Mean ± SD	Median (min-max)		Mean ± SD	Median (min-max)	p*
Total bilirubin (mg/dL)						
Pre-stenting	11.5 ± 8.1	9.2 (1.0 to 43.0)		9.7 ± 5.3	8.7 (0.3 to 22.2)	0.304
Post-stenting	6.0 ± 7.8	2.9 (0.3 to 41.8)		3.9 ± 4.5	2.6 (0.3 to 22.1)	
Difference	5.5 ± 8.0	4.9 (-9.0 to 38.2)		5.8 ± 6.6	5.3 (-13.7 to 20.8)	
Direct bilirubin (mg/dL)						
Pre-stenting	9.8 ± 6.6	8.4 (0.7 to 33.0)		8.5 ± 4.4	7.6 (0.2 to 18.8)	0.182
Post-stenting	5.1 ± 6.4	2.4 (0.2 to 29.7)		3.2 ± 3.7	2.3 (0.2 to 18.8)	
Difference	4.6 ± 6.5	4.3 (-9.5 to 28.7)		5.3 ± 5.3	5.0 (-10.9 to 18.1) 580	
Alkaline phosphatase (U/L)						
Pre-stenting	639.4 ± 438.5	505 (120 to 1911)		692.2 ± 439.1	(189 to 2358)	0.057
Post-stenting	493.3 ± 386.6	369 (63 to 2131)		437.1 ± 430.7	316 (66 to 2336)	
Difference	151.4 ± 431.4	113 (-769 to 1283)		224.9 ± 418.2	234 (-679 to 1310)	
Gamma-glutamyl transpeptidase (U/L)						
Pre-stenting	942.8 ± 674.9	811 (69 to 2855)		1029.1 ± 797.0	832 (75 to 4143)	0.154
Post-stenting	740.2 ± 779.1	440 (36 to 3848)		581.5 ± 664.7	346 (24 to 3468)	
Difference	200.9 ± 746.2	78 (-1347 to 2220)		396.0 ± 837.8	429 (-1022 to 2982)	
Aspartate aminotransferase (U/L)						
Pre-stenting	124.9 ± 69.9	114 (21 to 312)		130.3 ± 79.8	106 (25 to 382)	0.029
Post-stenting	93.8 ± 72.8	66 (16 to 307)		73.6 ± 95.1	39 (18 to 609)	
Difference	28.4 ± 86.8	27 (-175 to 292)		55.9 ± 103.3	36 (-350 to 278)	
Alanine aminotransferase (U/L)						
Pre-stenting	120.4 ± 83.7	99 (34 to 456)		128.7 ± 96.7	94 (31 to 507)	0.016
Post-stenting	69.6 ± 50.3	54 (14 to 321)		48.7 ± 26.1	41 (20 to 134)	
Difference	50.6 ± 88.3	28 (-72 to 376)		82.2 ± 94.7	41 (-48 to 390)	

SD, standard deviation.

* Nonparametric repeated-measures analysis of variance.

**Figure 1:**
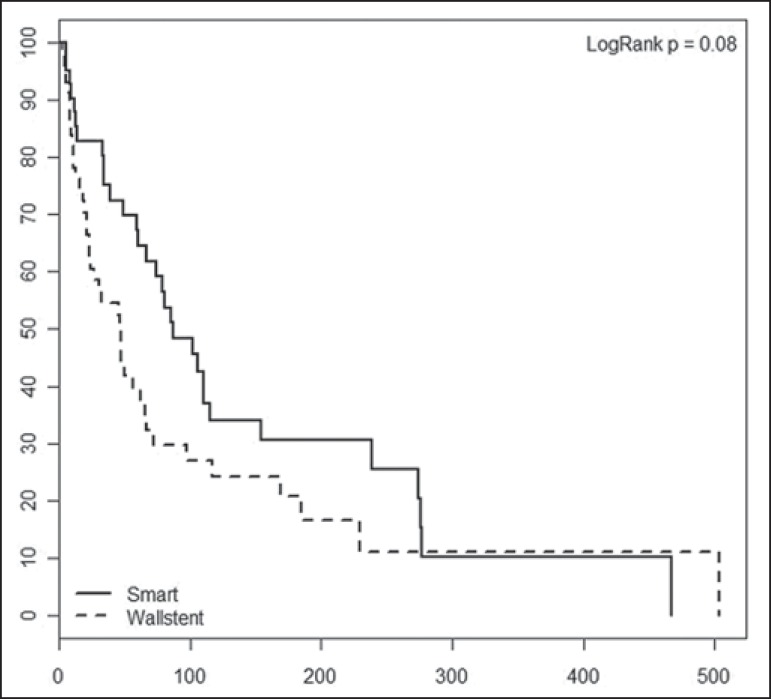
Occlusion-free survival curves according to the type of stent used (Kaplan-Meier analysis with log-rank test).

Stent occlusion was identified in 13 patients, 7 in the S.M.A.R.T. CONTROL group and 6 in the Wallstent group (*p* = 0.989). The number of stent occlusions at 30, 90, 180, and 360 days was 2, 1, 3, and 1, respectively, in the S.M.A.R.T. CONTROL group, compared with 2, 1, 2, and 1, respectively, in the Wallstent group. For the patients in both groups, occlusion-free survival rates were 67% at 30 days, 37% at 90 days, 25% at 180 days, and 10% at 360 days. When the survival curves were adjusted for age and level of biliary obstruction, there was no difference between the two groups. Occlusion-free survival rates at 30, 90, 180, and 360 days over time according to the type of stent used was 83%, 48%, 31%, and 10%, respectively, in the S.M.A.R.T. CONTROL group, compared with 57%, 30%, 21%, and 11%, respectively, in the Wallstent group. As can be seen in Figure 1, there was no statistically significant difference in survival curves in relation to the type of stent used (relative risk = 1.52; 95% confidece interval: 0.94–2.44; *p* = 0.085).

## DISCUSSION

Interventional procedures have been adopted at several radiological centers in Brazil^(13-24)^. Placement of self-expanding metallic stents in the setting of inoperable biliary malignancy is intended to improve the quality of life of affected patients via a minimally invasive procedure. In our series, the performance of the Wallstent was equivalent to that of the S.M.A.R.T. CONTROL stent in the percutaneous treatment of unresectable malignant biliary obstruction.

There are a wide variety of metallic stents currently available for the management of patients with biliary obstruction due to inoperable malignancy. The Wallstent is made of elgiloy and was one of the first stents developed for the treatment of malignant biliary strictures. To date, this has been the most widely studied stent, with satisfactory results having been reported in the literature^(25)^. However, the results of some studies have favored the use of stents made of nitinol because of their property of thermal shape memory and increased radial resistive force, which are associated with a theoretically lower risk of occlusion^(26)^. The S.M.A.R.T. CONTROL stent is made of nitinol, which has thermal shape memory, and has shown greater elasticity and resistance than have Wallstents in experimental studies^(27)^.

Few studies have comparatively analyzed different metallic stents to assess whether any one type is superior to the others in terms of long-term stent patency in the management of malignant biliary obstruction. Gandini et al.^(28)^ conducted a retrospective, nonrandomized study comparing the use of two different self-expanding metallic stents (Wallstent and Ultraflex) in 87 patients with unresectable malignant biliary obstruction and, as in our study, found that neither was superior to the other.

Studies evaluating the efficacy of using stents covered with polytetrafluoroethylene, polyethylene terephthalate, or polyurethane for the palliation of inoperable malignancies have reported no significant increase in survival compared with the use of uncovered stents^(29-31)^. However, it has been reported that patients treated with covered stents require fewer reinterventions and have a better quality of life^(30,31)^.

A retrospective, nonrandomized, comparative study involving 101 patients was conducted in order to examine the efficacy of the Niti-D biliary uncovered stent and the uncovered Wallstent for the palliative endoscopic management of malignant biliary obstruction^(32)^. The authors of that study found that stent patency tended to be longer in patients receiving the Niti-D stent (*n* = 41; 153 days) than in those receiving the Wallstent (*n* = 60; 124 days), although they reported no significant difference between the two stents (*p* = 0.204). In our study, better results were also observed in the S.M.A.R.T. CONTROL group, mainly for occlusion-free survival, although there was no statistically significant difference in survival curves in relation to the type of stent used (p = 0.085). We believe that the increased radial resistive force of the material employed in the S.M.A.R.T. CONTROL stent (nitinol) may have contributed to the lower rate of stent occlusion^(26)^.

Another issue to be considered is the high (28.3%) 30-day mortality rate observed in our study. That may be associated with the generally poor clinical condition of the patients, who all suffered from advanced, unresectable malignancy and most of whom (50.4%) already had diffuse metastases. A study involving 71 patients with obstructive jaundice due to solid malignancies was conducted to investigate the overall survival of patients after percutaneous transhepatic biliary drainage and showed that patients with poor performance status (Eastern Cooperative Oncology Group score > 2) have a dismal prognosis and should not undergo percutaneous transhepatic biliary drainage^(33)^. Currently, there are few effective treatment options for patients with unresectable malignant biliary obstruction, who have particularly poor survival characteristics. Therefore, although further survival improvement is unlikely to be obtained by stenting alone, we believe that this palliative treatment option meets its primary goal (i.e., to improve the quality of life of affected patients via a minimally invasive procedure), which appears to be independent of stent type.

The present study has some limitations. The retrospective nature of the study and the heterogeneity of the sample (with lesions of various histological types and at different sites) hinder the extrapolation of our results to other populations. In addition, because of the small number of stent occlusions, it was not possible to detect a statistically significant difference in occlusion-free survival rates between the two groups, despite the higher rates observed in the S.M.A.R.T. CONTROL group.

In conclusion, our results showed no clinically or statistically significant differences between Wallstents and S.M.A.R.T. CONTROL stents for the percutaneous treatment of unresectable malignant biliary obstruction. Both approaches appear to be safe and effective palliative therapies for affected patients, with good clinical results. Nevertheless, prospective randomized studies involving samples that are more homogeneous (comprising patients with lesions of a similar histological type, as well as similar obstruction sites and implantation techniques), are warranted in order to clarify this issue.
